# Floating–Harbor Syndrome: A Systematic Literature Review and Case Report

**DOI:** 10.3390/jcm13123435

**Published:** 2024-06-12

**Authors:** Wojciech Dobrzynski, Julia Stawinska-Dudek, Natalia Moryto, Dominika Lipka, Marcin Mikulewicz

**Affiliations:** 1 Department of Dentofacial Orthopaedics and Orthodontics, Division of Facial Abnormalities, Medical University of Wroclaw, 50-425 Wroclaw, Poland; marcin.mikulewicz@umw.edu.pl; 2 Student Scientific Group, Department of Dentofacial Orthopaedics and Orthodontics, Division of Facial Abnormalities, Medical University of Wroclaw, 50-425 Wroclaw, Poland; julia.stawinska-dudek@student.umw.edu.pl (J.S.-D.); natalia.moryto@student.umw.edu.pl (N.M.); dominika.lipka@student.umw.edu.pl (D.L.)

**Keywords:** Floating–Harbor syndrome, craniofacial abnormalities, dental anomalies, malocclusion, cephalometry, short stature

## Abstract

Floating–Harbor syndrome (FHS) is an extremely rare genetic disorder connected with a distinctive facial appearance, various skeletal malformations, delayed bone age, and expressive language delays. It is caused by heterozygous mutations in the Snf2-related CREBBP activator protein (SRCAP) gene. The aim of this paper is to describe the case of a 14-year-old male with FHS, referring to a review of the literature, and to collect all reported symptoms. In addition, the orthodontic treatment of the patient is described. For this, the electronic databases PubMed and Scopus were searched using the keyword “Floating–Harbor syndrome”. Similar to previous cases in the literature, the patient presented with short stature; a triangular face with a large bulbous nose; deep-set eyes and narrow eyelid gaps; a wide mouth with a thin vermilion border of the upper lip; and dorsally rotated, small ears. They also presented some less-described symptoms, such as macrodontia and micrognathia. Moreover, mild mental retardation, microcephaly, and delayed psychomotor development were found. On the basis of an extraoral, intraoral examination, X-rays, and CBCT, he was diagnosed with overbite, canine class I and angle class III, on both sides. To the best of our knowledge, orthodontic treatment of this disease has not been assessed in detail so far, so this is the first case.

## 1. Introduction

Floating–Harbor syndrome (FHS) is a rare genetic disorder characterized by a distinct constellation of mental, physical, and developmental features. This hereditary condition was first documented in 1973 [1,2,3].

The two American institutions at which the original cases were discovered are Boston Floating Hospital and Harbor General Hospital in California [3,4,5]. Although it is a less well-known disorder due to its rarity [1,2,4,5,6,7,8,9,10,11], understanding this syndrome is crucial for those who are affected and their family. A distinct collection of mental, physical, and developmental characteristics define FHS. In terms of appearance, it frequently manifests with distinctive facial characteristics such a triangular face [2,3,4,5,6,10,12,13,14], deep-set eyes [2,3,4,5,6,9,12,14,15], and a large nose [2,3,4,12,14]. Additionally, a noticeable delay in bone age [2,3,4,5,6,7,9,12] and small stature [1,2,3,4,5,6,7,9,10,11,12] present, and these features worsen with aging.

Developmentally, individuals with FHS may experience language delays [1,2,3,4,6,9,11,14], though their understanding of language and nonverbal communication skills are typically advanced. Their intellectual development is also affected, with most individuals showing mild to moderate intellectual disability [1,4,5,6,14]. The exact cause of FHS is a mutation in the SRCAP gene [2,4,5,6,9,10,12,16,17], a gene that encodes the SNF2-related CREBBP activator protein [2,4,9,12].

This mutation, which is normally not inherited, develops spontaneously either early in embryonic development or during the creation of reproductive cells. Although significant progress has been made in understanding the genetics of FHS, the condition’s rarity has made it difficult for researchers and healthcare practitioners to fully comprehend its impact and future treatment options. Numerous people with FHS and their families continue to have fulfilled lives in spite of these obstacles. With improvements in genetics and tailored medicine, there is fresh optimism that a better knowledge of FHS will result in more successful interventions, giving patients who have this condition a better future.

The aim of this study was to present a patient with FHS, their medical history, and orthodontic treatment plan, and to review the accessible scientific literature on FHS.

## 2. Materials and Methods

### 2.1. Diagnosis and Etiology

At the age of 13, the patient visited the Division of Facial Abnormalities within the Department of Orthodontics in Wroclaw for the first time. Deep bite and lower incisor retrusion were discovered during the physical examination. During this time, their side teeth were erupting. The boy had a removable appliance and was sent for diagnostics to see if fixed appliances could be used for orthodontic therapy. Their medical history states that during pregnancy, a rising disparity between the foetal head and trunk development levels was discovered. The baby boy, who weighed 1950 g and measured 47 cm at birth, was delivered via caesarean section at 35 weeks of pregnancy. According to cytogenetic analysis, a typical male karyotype was discovered. He was identified as having hypotonia and corpus callosum agenesis at birth. The patient has hypoplasia of the corpus callosum and mild mental retardation. They requires care from another person due to a lack of independence. A consulted psychiatrist diagnosed them with chronic tics and recommended psychological therapy, under constant psychological care also. During psychological therapy, the patient was found to be unable to recognize emotions. When asked a question, the patient reacted with a delay and postponement. In the opinion of a special educational centre, the patient needs constant support in their field of work during educational classes. The geneticist suspected Wolf–Hirschorn syndrome based on the child’s evaluation; however, genetic FISH investigation ruled this out and showed no abnormalities. A second diagnosis and follow-up appointments were advised. Specialists kept an eye on the boy’s health. The patient was described as having minor mental impairment, microcephaly, inadequate body weight, and delayed psychomotor development at the age of 5. In general, a facial dysmorphism study uncovered traits such as a triangular face, deep-set eyes, a wide nose, a low-hanging columella, low-set ears, macrodontia, and micrognathia. FHS was suspected based on the clinical signs and further tests. Molecular analysis was based on DNA sequencing method and covered a fragment of the 34th exon of the SRCAP gene (Val2395-Thr2500). The selected region of the SRCAP gene was analysed for the presence of Arg2435X and Arg2444X mutations. The patient presents clinical symptoms, suggesting the need for further genetic diagnostics. It is recommended that this diagnostic approach be expanded to include regions of the SRCAP gene that have not been previously examined. Additionally, it is worth considering the possibility of conducting an analysis of the coding region of the CREBP gene, for which the SRCAP gene acts as a co-activator. The 14-year-old patient recently showed up for an appointment at the Medical University of Wroclaw’s Department of Orthodontics, Department of Facial Abnormalities, following a CBCT scan.

**Figure 1:**
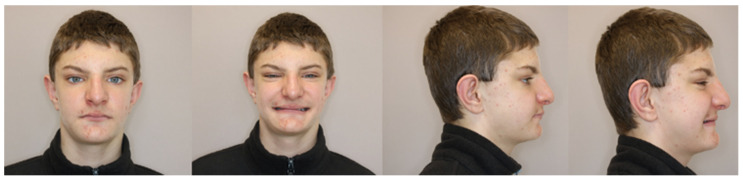
Extraoral photographs.

In an extraoral test, clinical examination revealed short stature [1,2,7] and dysmorphic facial features, such as a characteristic large bulbous nose [4], narrow eyelid gaps, a broad nasal septum, and chin protrusion. Their philtrum is short [3,10] and their mouth is wide with a lineal orientation and a thin vermilion border of the upper lip [12,14]. Moreover, an infantile method of swallowing was confirmed. The patient’s appearance is shown on extraoral photographs (Figure 1).

**Figure 2:**
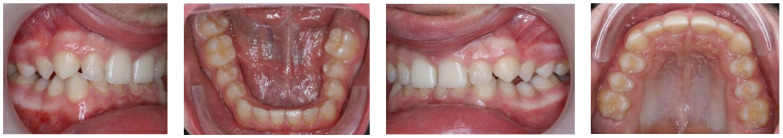
Intraoral photographs.

Intraoral examination revealed a shifted maxillary midline by approx. 2 mm to the left side, canine class I and angle class III on both sides, a deepened Spee curve, tilted upper and lower incisors, and vestibular surface abrasion of the lower incisors. The photographs taken during intraoral examination are presented as (Figure 2).

**Figure 3:**
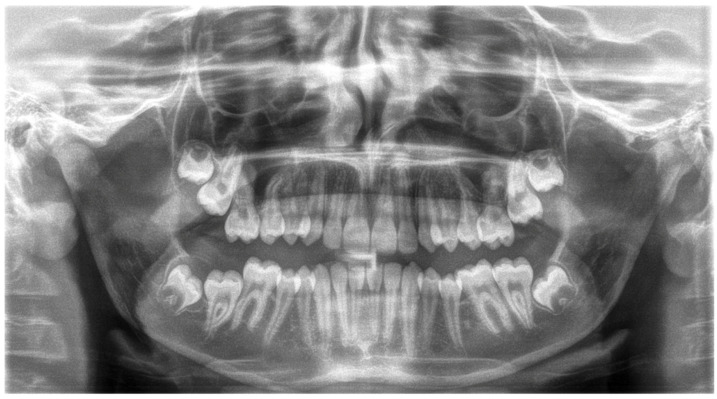
Orthopantomographic analysis.

**Figure 4:**
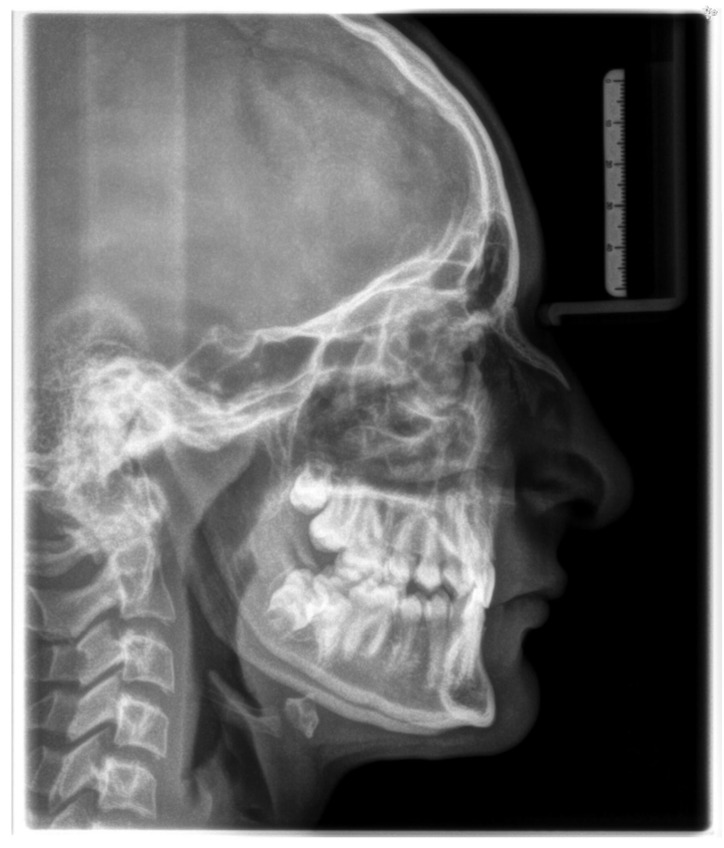
Lateral cephalometric radiograph.

**Figure 5:**
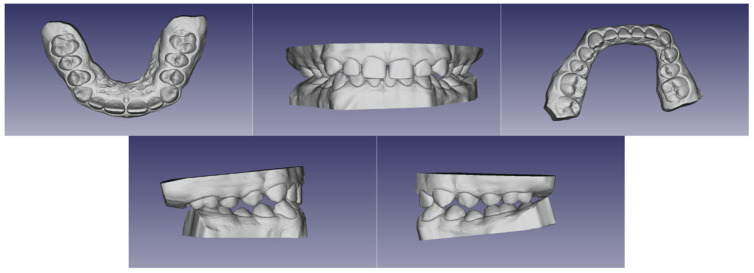
Intraoral scans.

In the first step of diagnostic imaging (September 2022), an orthopantomography evaluation (Figure 3) was performed. It demonstrated the presence of all permanent teeth and four buds of wisdom teeth. The cephalometric radiograph is shown in Figure 4, and the outcome of the cephalometric analysis is presented in Table 1. Furthermore, the intraoral scans were taken with a 3Shape TRIOS 4 scanner (3Shape Trios A/S, Copenhagen, Denmark) (Figure 5).

### 2.2. Treatment Objectives

**Figure 6:**
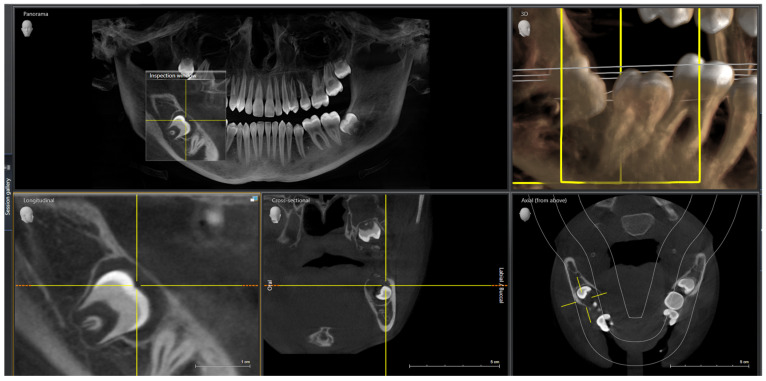
Cone beam computed tomography (CBCT).

During consultation the treatment plan was presented, which included the installation of fixed braces in the upper arch and lower lingual to verticalize the lower molars, prevent tipping [18], and strengthen the anchorage when bringing tooth 47. It was advised that brackets with high torque be placed in the upper arch, and brackets with a low torque in the lower arch to reduce deflection. At the bottom, intrusion of the incisors might be necessary. Additionally, the extraction of tooth 48 was planned because of the follicular cyst that is visible on the CBCT scan (Figure 6).

After accepting the treatment plan, the patient was referred for extraction of tooth 48, which took place in the Dental Surgery Department of Wrocław Medical University. The patient was under infiltration and conduction anaesthesia. The surgeon performed a mucoperiosteal flap in envelope technique to expose impacted teeth 47 and 48. Tooth 48 was removed by separation and the odontogenic cyst was enucleated. The specialist glued the button with a hook on the exposed tooth 47.

Since the extraction, the patient has been under the care of the Orthodontic Department and currently is being treated with fixed braces in the upper arch. MBT brackets were placed with high torque brackets on the upper incisors and TriColore on the other teeth. The orthodontic treatment of patients with Floating–Harbor syndrome does not differ from standard treatment methods in completely healthy people. There was no tendency for increased spontaneous resorption of the tooth roots, as observed in Turner syndrome

### 2.3. Literature Review

**Figure 7:**
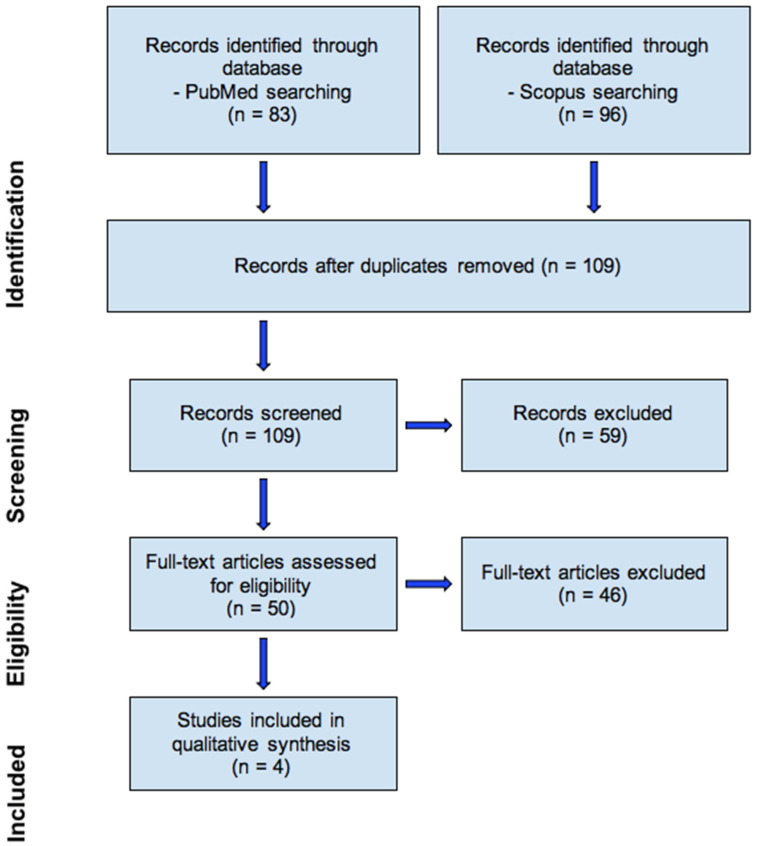
Prisma flow diagram.

The electronic databases PubMed and Scopus were searched from 2003 to 2023. The language of the articles was restricted to English. The following keyword was used for the search: *Floating–Harbor syndrome*. In total, 83 articles from PubMed and 96 articles from Scopus were initially included in this study. After removing duplicated papers, 109 articles were chosen. Among them, case report formats were distinguished (50) as an inclusion criterion, 4 of which contained information about dental abnormalities and did not involve animal studies. Exclusion criteria were articles that were not case reports, not about dental abnormalities, and those that were animal studies. The results are shown in the PRISMA flow diagram (Figure 7).

## 3. Results

According to the specified literature (Figure 7), some characteristics are summarized in the table (Table 2). It was noticed that all of the patients were born of healthy parents. Nevertheless, the hereditary nature of the syndrome cannot be ruled out. Four out of five patients are boys but based on the literature generally available, no gender trends in patients can be identified. All of the analysed cases reported mild mental retardation and characteristics specific to FHS facial dysmorphism like a bulbous nose, thin upper lip, or wide columella. In at least one previous article (also in this one), there was a description of a boy with NSBa above the normal cephalometric measurements, which indicates a flattened base of the skull that could be due to the retrognathic face. However, there are insufficient studies confirming the consistency of this feature in patients with FHS. In this case, the patient had intraoral abnormalities such as overbite, class III molar relationship, or hypodontia. However, the cases are not sufficiently described to conclude that such changes are specific to FHS. Malocclusions can also occur alongside other diseases, and some patients in the literature exhibit a full set of permanent teeth. The topic of intraoral defects in FHS is still poorly understood and requires many analyses and further research to systematize the current knowledge.

## 4. Discussion

Floating–Harbor syndrome is a rare genetic disorder with specific symptoms, including face, skeletal, intellectual speech, and systemic abnormalities. The triad of the most characteristic symptoms are short stature with a significantly delayed bone age, retarded speech development, and an abnormal and triangular face [11]. The syndrome is caused by heterozygous mutations in the Snf2-related CREBBP activator protein (SRCAP) gene [2]. According to data from 2021, more than 100 cases of FHS have been reported worldwide [7], with the first reported in 1973 [3].

### 4.1. Face

The characteristics of FHS include dysmorphic facial features, such as a small triangular face, abnormally long eyelashes, broad deep-set eyes, a low hairline [5], a large bulbous nose that is narrow at the root and broadens to the tip [6], and low-set dorsally rotated ears [14]. Patients’ mouths are wide, with a lineal orientation at rest or when smiling [10], and a thin vermilion border of the upper lip [12]. The philtrum is short and the columella is wide and low-hanging [12]. Dental anomalies are sparse (malocclusion, hypoplastic jaw, high-arched palate, increased spacing, agenesis of mandibular incisors, supernumerary teeth, and oligodontia) [2].

### 4.2. Body

Among the symptoms, we also distinguished skeletal anomalies such as brachydactyly, fifth finger clinodactyly (which are most common) [1], short phalanges [2], cone-shaped and sclerotic epiphyses [12], spine disorders [2], and a short neck [6]. Most of the cases present proportional short stature because of their significantly delayed bone growth in the first decade of life [5].

### 4.3. Speech and Intellectual Functioning

Characteristic symptoms include expressive language delay and deficits [12] as well as speech impairment [4], which may be related to immobility of the palate [3]. A high-pitched and hypernasal voice [6] in addition to conductive hearing loss [16] are usually noted in individuals with FHS. Intellectual disability is mild to moderate [9].

### 4.4. Other Abnormalities

Endocrine studies of growth hormones, somatomedin C test reults, and thyroid function show abnormal results [3], and hirsutism is noticed in some cases [1,3,14]. Puberty development appeared to be normal among these patients [12]. Occasionally, there were some gastrointestinal, cardiac (congenital heart defects), and genitourinary (for example hydronephrosis) disorders [7]. Some features and their frequency based on the referenced literature are presented in the table (Table 3).

This article aims to outline the patient profile, medical history, and orthodontic treatment progression of FHS. To our knowledge, detailed assessments of orthodontic treatments of this condition are lacking, making this the inaugural case study. FHS is detected in early childhood and affects both sexes [1,2,4,5,7,9,11,12,14]. There are cases of premature birth [11,12,14] or low weight [1], but there are also cases without perinatal complications [2,4,5,7,9,12]. Short stature, which is a symptom included in the characteristic triad, is noticed in every case [1,2,4,5,7,9,11,12,14]. Apart from mild mental retardation confirmed at the age of 5 (also described in most of articles [1,2,4,5,9,11,14]), there is also a characteristic facial appearance, which is particular to this disease [1,2,4,5,7,9,11,12,14]. The patient’s dysmorphic features, such as a triangular face [1,4,5,7,9,11], bulbous nose [4,5,6,7,11,14], low-hanging columella [1,6,9,11], wide mouth with lineal orientation [4,7,11], short philtrum [4,5,9,11], and deep-set eyes [4,5,6,7,9,11], were noticed in some cases. Micrognathia, macrodontia, and overbite are characteristic in our patient. Dental abnormalities are often described in FHS [1,2,4,5,7,9,11,14]. In addition, patients often struggle with other systemic diseases like gastrointestinal [7] or cardiac problems [4], and they remain under the care of specialists.

Diagnosis is not easy. FHS symptoms and symptoms of other specific diseases such as Dubowitz syndrome, Rubinstein–Taybi syndrome (mutations in the CREBBP or EP300 genes), 3 M syndrome, and Russell–Silver syndrome (abnormalities affecting certain genes on chromosomes 7 or 11) can overlap [1]. A diagnosis of FHS is suspected when individuals show typical clinical findings, and it can be confirmed by sequencing SRCAP [2].

## 5. Conclusions

Floating–Harbor Syndrome is an exceedingly infrequent condition. The aetiology of this condition is still not clear. Opinions exist stating that the syndrome is caused by spontaneous mutations or is inherited in an autosomal dominant manner [19]. The mandatory clinical features for reaching a diagnosis include characteristic facial features and language delay [20]. The disease is severe because due to the synchronously occurring diverse symptoms in different systems. Dental anomalies are sparse but varied. They relate to the teeth, jaw, palate, or even lips [2]. While diagnosing a patient suspected to have FHS, OPG (orthopantomogram) and cephalometry are essential. FHS is a genetic illness, so its treatment is symptomatic. Older patients, such as the boy studied in this case report, are recommended to wear a fixed appliance to correct defects.

## Figures and Tables

**Table 1:** Cephalometric measurements of the 14 y.o. patient.

Description	Cephalometric Measurements	Value	Mean	SD
**Maxilla to Cranial Base**	SNA	78.67	82.00	3.00
	NL-NSL	11.29	8.00	4.00
**Mandible to Cranial Base**	SNB	79.03	80.00	3.00
	ML-NSL	25.82	28.00	5.00
**Maxilla to Mandible**	ANB	−0.36	3.00	2.50
	WITS [mm]	−2.40	0.00	2.00
	ML-NL	14.53	20.00	7.00
**Maxillary Dentition**	U1-NA [mm]	1.50	3.70	2.00
	U1-NA	16.32	21.00	4.00
**Mandibulary Dentition**	L1-NB [mm]	−1.98	3.80	5.00
	L1-NB	2.44	24.00	4.00
**Soft Tissue**	Nasio-labial angle	89.72	110.00	7.00
	SNPg	81.49	81.00	3.00
	N-S-Ba angle	138.49	132.00	4.00
	Gn-tgo-Ar angle	133.52	122.00	7.00
	NB-H angle	6.09	9.00	3.00
	U1-L1	161.60	133.00	8.00
	Pg-NB [mm]	4.91	2.30	2.00
	N-Sp’/Sp’-Gn [%]	88.21	80.00	7.00

**Table 2:** Frequency of clinical characteristics present in previous studies and comparison with our patient.

Characteristics	Previous Studies	This Study
sex	3 boys, 1 girl	a boy
mental retardation	4/4	+
speech disorders	3/4	+
facial dysmorphism	4/4	+
malocclusion	4/4	+

“+”—the presence of a given feature.

**Table 3:** Systematic review.

Article	Number of Patients	Patient’s Age (years)	Patient Gender	Birth Complications	A	B	C	D	E	F	G	H
[2]	1	14	Male	-	+	+	+	+	+	+	+	+
[5]	1	7	Male	-	+	+	+	+	−	+	+	+
[7]	1	7 y * 9 m **	Male	-	+	+	+	+	−	+	?	+
[11]	1	5.5	Female	Born at 30 weeks; foetal distress	+	+	+	+	−	+	+	−
[1]	1	5	Male	Perinatal asphyxia, MSAF, low weight	+	+	+	+	−	+	+	+
[12]	7	2 y 2 m 13 y 7 m	5 Males 2 Females	1/7: born at 32 weeks;	7/7	7/7	5/7	6/7	2/7	?	5/7	7/7
[4]	1	2.5	Male	-	+	+	+	+	+	+	?	+
[9]	12	1–9 y 2 m	6 Males 6 Females	-	12/12	7/7	9/12	12/12	?	7/12	11/12	7/12
[18]	1	6 y 11 m	Female	preterm	+	+	+	+	−	+	+	−

Short stature—A; retarded speech development—B; skeletal abnormalities—C; facial dismorphy—D; clefts—E; dental abnormalities—F; intellectual disability—G; systemic symptoms—H; * y—years, ** m—months. “+”—the presence of a given feature, “−”—a given feature is absent.

## Data Availability

Not applicable.
